# Cannabinoid Quinones—A Review and Novel Observations

**DOI:** 10.3390/molecules26061761

**Published:** 2021-03-21

**Authors:** Natalya M. Kogan, Maximilian Peters, Raphael Mechoulam

**Affiliations:** Institute for Drug Research, Medical Faculty, Hebrew University, Jerusalem 91120, Israel; maximili.peters@mail.huji.ac.il (M.P.); raphaelm@ekmd.huji.ac.il (R.M.)

**Keywords:** cannabinoid, quinones, anti-cancer, structure-activity relationship

## Abstract

A cannabinoid anticancer para-quinone, HU-331, which was synthesized by our group five decades ago, was shown to have very high efficacy against human cancer cell lines in-vitro and against in-vivo grafts of human tumors in nude mice. The main mechanism was topoisomerase IIα catalytic inhibition. Later, several groups synthesized related compounds. In the present presentation, we review the publications on compounds synthesized on the basis of HU-331, summarize their published activities and mechanisms of action and report the synthesis and action of novel quinones, thus expanding the structure-activity relationship in these series.

## 1. Introduction

Quinones of various chemical families have biological activities [1,2,3], and both natural and synthetic quinones are widely used as drugs [4,5]. Anthracyclines, a large group of quinonoid compounds produced by different strains of Streptomyces, exert antibiotic and antineoplastic effects, and are used to treat some forms of cancer [5]. The best-known members of this family are daunorubicin and doxorubicin, the first identified anthracyclines [6]. Mitomycin C and streptonigrin produced by Streptomyces and the synthetic epirubicin and mitoxantrone are used as anticancer drugs [7]. Although these and other quinonoid compounds are effective in the treatment of many different forms of cancer, their side effects—the most severe being cumulative heart toxicity—limit their use [8,9,10]. The development of quinonoid compounds that display antineoplastic activity, but are less toxic, is a major therapeutic goal.

A large number of cannabinoids have been synthesized and tested in various in vitro and in vivo assays, including models of several diseases [11,12,13,14]. A cannabinoid anticancer p-quinone, HU-331 (HU standing for Hebrew University), was synthesized by our group back in 1968, to address a chemical question of which cannabinoids give a purple color when hashish extract is treated by 5% aqueous KOH in methanol (the Beam Test) [15]. Much later, in the 1990s, this compound was suggested to be a hepatic metabolite of CBD [16,17]. It was also found to decrease the microsomal content of a monooxygenase CYP450 enzyme, an effect prevented by co-incubation with glutathione (GSH) or cysteine, presumably by the radical-scavenging action of these antioxidants [16]. In 2001, while searching for possible anticancer cannabinoids, we synthesized HU-331 again, this time as an attempt to combine the quinonoid anticancer moiety with cannabinoid molecules, which are mostly non-toxic. Cannabinoids are known to possess distinct pharmacokinetic and distribution patterns than the known quinonoid anticancer drugs. HU-331 was shown to have very high efficacy against human cancer cell lines in-vitro and against in-vivo grafts of human tumors in nude mice, especially in colon carcinoma HT-29 and in Raji lymphoma [18]. The mechanism of action of HU-331 in cancer cells was found to be the inhibition of topoisomerase IIα, without topoisomerase poisoning [19]. It caused neither cancer cells apoptosis nor cell cycle arrest. It did not affect the intracellular pathways of p38MAPK, pERK, c-jun or p53. 

The effect of HU-331 as inhibitor of topoisomerase IIα seemed very selective [19]. This finding was later also supported by Dewesee’s group [20] and Marson’s group [21]. In addition, we found it to possess anti-angiogenic effects [22], and unlike most other quinones, HU-331 was not cardiotoxic [23,24]. However, it seems that in different cells the mechanism of action of HU-331 may vary from the one found by us. The first mention of a different mechanism was by Usami et al. in 2008, who suggested that HU-331 generated reactive oxygen species (ROS) during mouse hepatic microsomal metabolism in the presence of NADPH [25]. Another mechanism was suggested by Wu and Jan (2010), who found that HU-331 treatment significantly enhanced splenocyte apoptosis in a time- and concentration-dependent manner. A gradual decrease in the levels of cellular thiols and glutathione was detected in HU-331-treated splenocytes. The apoptosis and thiol diminishment induced by HU-331 were abrogated in the presence of thiol antioxidants, including N-acetyl-L-cysteine and N-(2-mercaptopropionyl) glycine, while the non-thiol antioxidants catalase and pyruvate were ineffective [26]. The search for possible additional mechanisms of HU-331 still continues, with a recent publication showing that HU-331 modulates a peroxisome proliferator-activated receptor gamma (PPARγ)-based activity [27].

**Figure 1:**
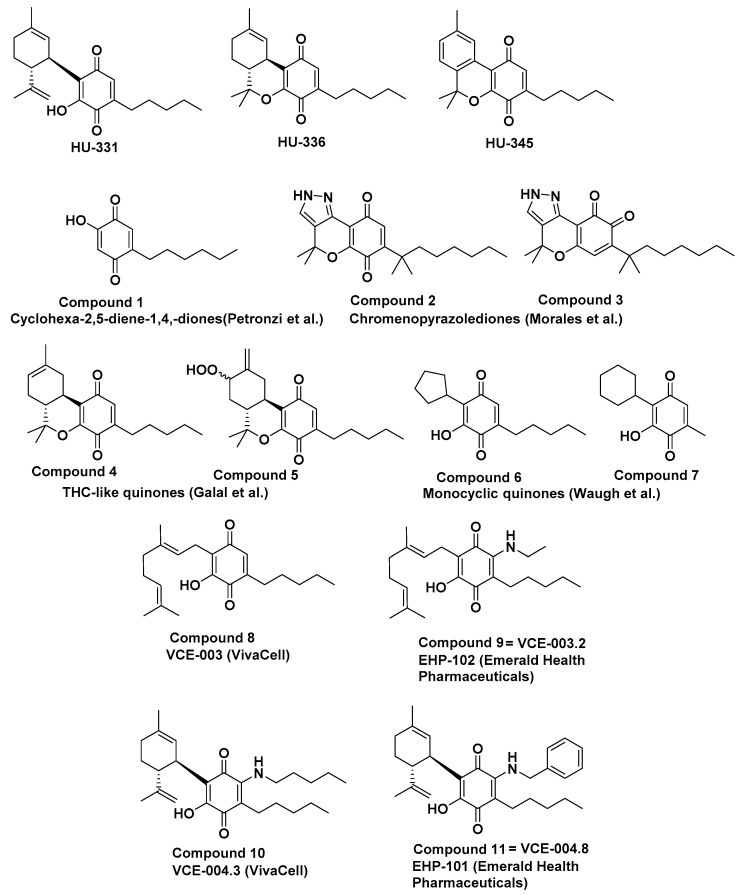
HU-331-like compounds.

After our reports on HU-331, several groups reported syntheses of novel cannabinoid-like quinones. In 2013, Petronzi et al. synthesized some novel quinones, related to HU-331. In the most potent molecule of this series (Compound **1**, Figure 1), the side-chain is elongated to six carbon atoms (instead of five carbon atoms as in HU-331), and is shifted to position 2 of the quinone ring, leaving position 3 open. The terpene ring that is present in position 6 of HU-331 is deleted. This molecule was found to be active against some cancer cell lines, especially M14 melanoma. The mechanism of action of this new molecule differs from that of HU-331: a time-dependent pro-apoptotic activity on human melanoma M14 cell line was seen, mediated by ROS, caspases activation and poly-(ADP-ribose)-polymerase (PARP) protein cleavage. Neither topoisomerase I nor II were involved [28].

That same year, Jagerovic’s group synthesized p-quinones from their previously-discovered cannabinoid-like chromenopyrazoles [29]. Some of them showed significant affinity in the sub-micromolar range to both CB1 and CB2 receptors. The most active compound was named PM49 (Compound **2**). These compounds were more effective in prostate carcinoma than in hepatocellular carcinoma cells. The mechanism of action of PM49, however, differed from that of HU-331. The CB1 antagonist rimonabant, as well as the PPARγ antagonist GW 9662 and the antioxidant N-acetyl cysteine (NAC), were able to significantly reduce the inhibition of cell viability induced by PM49. It was found to cause cancer cells apoptosis, also unlike HU-331 [30]. Later (in 2015), the same group continued their research on compounds related to the chromenopyrazoles quinones, but in the ortho configuration. All the novel cannabinoid quinone derivatives displayed potent growth inhibitory effects on triple-negative MDA-MB-231 cells with low micromolar IC50 values. Compound **3**, selected for further development, was also able to effectively reduce the growth of triple-negative breast cancer xenografts in vivo [30].

In 2018 ElSohly’s group reported research with cannabinoid anticancer quinones based on tricyclic THC-like cannabinoids. The introduction of the p-quinone moiety by photooxygenation of THC and its derivatives led to a loss of affinity to the cannabinoid receptors CB1 and CB2. The quinone derivatives exhibited anticancer and marked antimicrobial activity. One of these (Compound **4**) was a potent anti-cryptococcal agent. The best agent against S. aureus was Compound **5**; both compounds were antiproliferative in different cancer cell lines, while others showed anti-candidal activity [31].

Recently, Marson’s group added an additional piece to the puzzle of cannabinoid quinones and their action on topoisomerase II, by synthesizing about 15 derivatives related to HU-331, and assaying them for topoisomerase inhibition and cancer cell growth inhibition (mostly DU-145 prostate carcinoma) [21]. The most active derivatives were those with cyclohexyl and cyclopentyl substitution at the place of limonene in HU-331, with no substituent in position 2 of the quinone ring, and with pentyl or methyl groups in position 3 (Compounds **6** and **7**).

Casares et al. recently published that the methoxylated HU-331 derivative has dual activity as NRF2 activator and BACH1 inhibitor [32]. The axis NRF2-BACH1 has anti-inflammatory and antioxidant properties that could be exploited pharmacologically to obtain neuroprotective effects. Indeed, this derivative provides neuroprotection in cell models of relevance to Huntington’s disease [32]. 

Recently, a Polish group investigated the anti-cancer activity of several topoisomerase inhibitors, amongst them HU-331, combined with cisplatin. All the investigated topoisomerase II inhibitors in combination with cisplatin efficiently killed U-87 cells. The most potent cytotoxic effect was observed for cisplatin with HU-331 [33].

In a different line of cannabinoid quinone research, back in 2012, researchers in the VivaCell company, in collaboration with colleagues in two Spanish universities, searched for additional biological activities, different from cancer cell growth inhibition. The quinone of cannabigerol, named VCE-003 (Compound **8**), showed prominent anti-inflammatory activity, protected neuronal cells from excitotoxicity, activated PPARγ transcriptional activity and inhibited the release of pro-inflammatory mediators in LPS-stimulated microglial cells [34]. It also ameliorated the symptoms associated with TMEV (Theiler’s murine encephalomyelitis virus) infection, a model of multiple sclerosis (MS), leading the authors to assume that cannabinoid quinones may be useful in the treatment of MS and other neuroinflammatory disorders [34]. In a later experiment (in 2014), the same group showed that VCE-003 ameliorated the neurological defects and the severity of MOG-induced EAE (myelin oligodendrocyte glycoprotein-induced experimental autoimmune encephalomyelitis) in mice through CB2 and PPARγ receptor activation, at least partially, as antagonists of both these receptors partially blocked VCE-003 activity [35]. Later, in 2016, this compound was modified to VCE-003.2 (Compound **9**), which is a representative of a second generation of cannabigerol quinone non-electrophilic derivatives, possessing the same anti-inflammatory and neuroprotective effect [36]. In line with the authors’ initial observation of the action of cannabinoid quinones in neurodegenerative diseases, they assayed VCE-003.2 in Huntington’s models of striatal neurodegeneration induced by quinolinic acid and 3-nitropropionic acid. VCE-003.2 prevented medium spiny DARPP32+ neuronal loss in these Huntington’s-like disease mice models, improving motor deficits, reactive astrogliosis and microglial activation, inhibiting the upregulation of proinflammatory markers and improving antioxidant defenses in the brain [36]. In further work on this compound, in 2018, it was also shown to protect the mice against Parkinson’s disease development. The administration of VCE-003.2 to lipopolysaccharide (LPS)-lesioned mice (a model for Parkinson’s disease) attenuated the loss of tyrosine hydroxylase (TH)-containing nigrostriatal neurons, and in particular, the intense microgliosis caused by LPS in the substantia nigra [37]. In a very recent study (2021), VCE-003.2, as well as cannabigerolic acid quinone and its sodium salt, were found to be active in 6-OHDA lesioned mice, an additional model of Parkinson’s disease [38].

The analysis by quantitative Polymerase Chain Reaction (qPCR) of proinflammatory mediators such as tumor necrosis factor-α (TNF-α), interleukin-1β (IL-1β), and inducible nitric oxide synthase (iNOS) in the striatum showed that they were markedly elevated by the LPS lesion, and strongly reduced by treatment with VCE-003.2. The effects of VCE-003.2 in LPS-lesioned mice implied the activation of PPARγ receptors, as they were attenuated when VCE-003.2 was co-administered with a PPARγ inhibitor [37]. However, the in-vitro effects were not attenuated by PPARγ inhibitors, suggesting additional possible mechanisms [37]. Oral administration of VCE-003.2 also generally helped in a Parkinson’s model, though in higher doses than the previous intraperitoneal treatment [39]. In an additional neurodegenerative disease model with SOD1G93A mutant mice (a model of Amyotrophic Lateral Sclerosis, ALS), treatment with VCE-003.2 improved most of the neuropathological signs such as weight loss and neurological deterioration, which was associated with a marked loss of spinal cholinergic motor neurons and glial reactivity, thus indicating the effectiveness of this compound also for ALS treatment [40]. This molecule, which is being developed by Emerald Health Pharmaceuticals, is now named EHP-102, and was granted both FDA and EMA orphan designation for the treatment of Huntington’s disease.

In parallel with CBG-quinones derivatives, the same research group worked also on CBD-quinones. VCE-004.8, a benzyl-amino derivative of HU-331 (Compound **10**), was found to possess a dual agonist activity on PPARγ and CB2 receptors, and anti-fibrotic activity in scleroderma models [41]. Another derivative, VCE-004.3, a pentyl-aminoquinone derivative of HU-331 (Compound **11**) showed the same activity [42]; however, the company apparently preferred to move forward with the VCE-004.8 derivative. It was reported to attenuate adipogenesis, to prevent diet-induced obesity [43], to activate the hypoxia inducible factor (HIF) pathway, to prevent demyelination, axonal damage, immune cell infiltration in EAE and to be effective in TMEV models of MS [44]. Furthermore, VCE-004.8 was formulated for oral administration, the anti-fibrotic activity of which was the same as an intraperitoneal injection [45]. In a recent publication (2020), this oral formulation was also found to be active in the MS models [46]. Now this molecule, which is being developed by Emerald Health Pharmaceuticals, is named EHP-101, and was granted both FDA and EMA orphan designation for the treatment of scleroderma. At present, it is in Phase II clinical trials on Diffuse Cutaneous Systemic Sclerosis, a subtype of systemic scleroderma. 

The structure-activity relationship (SAR) of the cannabinoid quinones is still not very clear. Different molecules possess different mechanisms of action, especially those of the original HU-331 line, and those in which position 2 is substituted with amine derivatives.

Now we report the syntheses of additional compounds in the cannabinoid quinone series, which presumably may help to shed more light on the SAR and the mechanism of action of HU-331 and related compounds.

## 2. Results

### 2.1. Chemistry

In our original reports [15,18], the oxidation of cannabidiol (CBD) by air in petroleum ether in the presence of 5% aqueous potassium hydroxide in ethanol over 3 h at 0 °C led to the formation of the HU-331 in about a 20% yield. We have now found that a slight change in the reaction conditions—stirring the reaction in O_2_ atmosphere instead of air, and adding a few drops of DMSO to the reaction mixture—enhances the yield to about 50%. 

**Figure 2:**
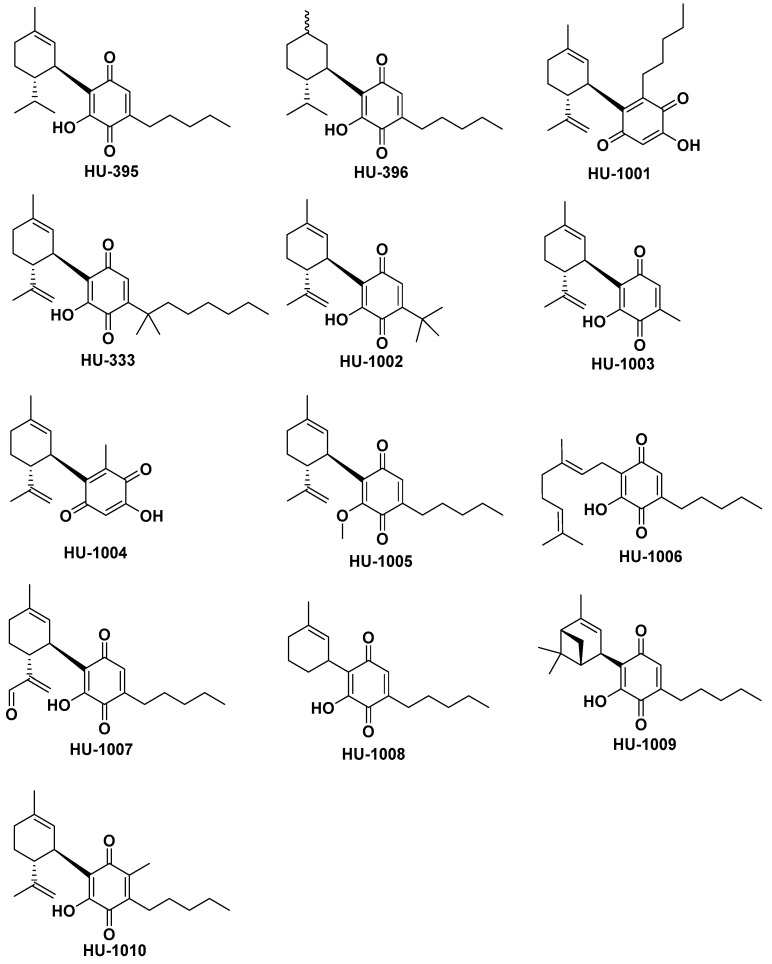
HU-331-like compounds synthesized in this study.

In our original evaluations of the anticancer activity of cannabinoid quinones [18] we observed that the quinone of CBD (HU-331), a two-ring compound, was much more active than the quinone derivatives of the three ring compounds, tetrahydrocannabinol (THC) (HU-336) and cannabinol (CBN) (HU-345) (Figure 1). Hence, we proceeded with synthesizing HU-331-like compounds in which ring B is open (Figure 2).

First, we investigated whether the double bonds in the terpenoid ring have any effect on the activity of HU-331. For this purpose, CBD was reduced with platinum oxide over hydrogen to obtain 8,9-dihydrocannabidiol and 1,2,8,9-tetrahydrocannabidiol. Their quinonoid derivatives (HU-395 and HU-396) were then synthesized, by the same method as HU-331 from CBD.

In the synthesis of CBD by the coupling of p-menthadienol with olivetol, two products are obtained—CBD (yield around 30%) and a compound in which the terpenoid ring is connected to the phenolic ring on the carbon between the phenol and pentyl side chain (yield around 60%), known as abnormal cannabidiol (abnCBD) [47]. To investigate whether the position at which the rings are attached to each other is important for the anticancer activity of these compounds, the quinone of abnCBD (HU-1001) was synthesized by the same method as the quinone HU-331.

The replacement of the pentyl side chain (which is present in most plant cannabinoids) with a dimethylheptyl side chain has been noted to lead to compounds with higher potency than that of the natural compounds in different bioassays [48,49,50]. Hence, we synthesized the quinone of dimethylheptyl-cannabidiol (CBD-DMH) (HU-333) from CBD-DMH by the same method as HU-331 from CBD. For SAR reasons, we also prepared and assayed the novel quinones of CBD-like compounds in which the side chain was tert-butyl (HU-1002), methyl (HU-1003), and in the abn-CBD series, the methyl homolog (HU-1004). The tert-butyl homolog was prepared in order to observe whether substitution on the first carbon of the side chain—as we see in CBD-DMH (HU-333)—will elevate activity.

As HU-331 differs from the quinone of THC by having ring B open and quinone hydroxyl group free (see Figure 1), we decided to investigate whether a free hydroxyl on the quinone ring is essential for the anticancer activity (HU-331 compared to HU-336). Hence, the hydroxyl group on the quinone ring of HU-331 was blocked by methylation, leading to quinone HU-1005. 

To investigate the role of ring A in the anticancer activity of the quinone, we synthesized and investigated the quinone (HU-1006) formed from cannabigerol (CBG), in which the hydroxyl on the quinone ring is free and neither A nor B rings are present. 

In different models of CBD activity, oxidations on ring A produce more powerful molecules. Hence, we synthesized the 9-oxo derivative (HU-1007). To investigate whether the isopropenyl chain, attached to position 4 of the cyclohexene ring in CBD-331 plays any role in its anticancer activity, a CBD-like compound with cyclohexyl instead of terpenoid ring was synthesized by coupling 1-hydroxy-1-methyl-cyclohex-2,3-ene to olivetol. The quinone (HU-1008) of this compound was synthesized from it by the same oxidation method as HU-331 from CBD. To investigate whether the isopropenyl chain has to be free or can be connected as a bridge in ring A, the quinone HU-1009 of a known bicyclic pinene-derived compound was prepared by the standard oxidation procedure. 

In the first attempts to synthesize HU-331, the dimer of this compound (where two HU-331 units are connected through 2′ position of the quinone ring) was formed during the reaction. It possessed no anticancer activity. Using the new reaction conditions, we do not obtain the dimer during the reaction; however, it is quite rapidly formed if HU-331 is left dissolved at room temperature. By preventing this dimerization, it should presumably be possible to prolong the action of HU-331 in the cells, thus improving its anticancer activity. A CBD derived quinone in which the position 2′ of the quinone ring is blocked by a methyl group, was prepared by oxidation of the known [51] 2′-methyl CBD to quinone HU-1010.

### 2.2. Biological Evaluation

All the aforementioned compounds were assayed for their ability to cause cell death in two human cancer cell lines which were found to be the most sensitive for HU-331 from a panel of numerous cancer cell lines [18]—Jurkat (T-cell lymphoma) and Raji (Burkitt’s lymphoma). All of the compounds’ effects are significantly different from vehicle-treated group. The IC_50_ values are presented in Table 1.

The results presented in Table 1 contribute to our understanding of cannabinoid quinone SAR. With the hydrogenated derivatives HU-395 and HU-396, we noted that HU-396 is slightly less active and HU-395 slightly more active than HU-331, indicating that the double bonds in the terpenoid part of the molecule play only a minor role in its ability to kill cancer cells. On the contrary, the position at which rings A and C are attached to each other seems to be of great importance, as the “abnormal” version of HU-331, namely HU-1001, does not affect these cells. In fact, it is one of the least potent compounds. 

As in numerous bioassays cannabinoids with dimethylheptyl side chain on the aromatic moiety are more potent than the natural pentyl-chain compounds, we expected HU-333 to be more active than HU-331. However, it was significantly much less active in both cell lines, indicating that in this case, the SAR differs from the SAR usually observed for cannabinoids. The t-butyl derivative, HU-1002, acted slightly better than DMH, but significantly worse than HU-331, showing that a bulky substituent on the aromatic moiety interferes with the activity. The short-chained derivative HU-1003 was slightly less potent than HU-331 showing that possibly there is some minimal chain length required for activity. Thus, although the DMH usually causes molecules to better penetrate the cell, which should improve its activity, here it probably interferes with quinones binding to their intracellular target, thus making the molecule less active.

The quinone derived from the methyl homolog of abnCBD (HU-1004) showed significantly lower activity in both cell lines in either the Jurkat or the Raji cells. 

As the quinone of THC (HU-336), in which there is no free hydroxyl on the quinone ring acted much worse than HU-331, we expected HU-1005, in which the hydroxyl on the quinone ring is blocked by methyl, to be much less active than HU-331 as well. However, HU-1005 acted nearly as well as HU-331 on cancer cells, indicating that it does not matter for the anticancer activity whether the hydroxyl group is free or blocked. Thus, the second major difference between THC quinone (HU-336) and CBD quinone (HU-331), the closed ring B in the THC quinone, which prevents the free rotation of the rings, is apparently inappropriate for activity.

The importance of ring A is seen from the fact that HU-1006, which does not possess this ring, acts significantly worse than HU-331 on both cell lines. 

As previously found for other CBD-like molecules, when the methyl group in the C9 position in the terpene moiety is oxidized to an aldehyde, leading to HU-1007, the activity is enhanced.

HU-1008, which has no propenyl attached to position 4 on the ring A, acts significantly worse than HU-331 in both cell lines, indicating that the isopropenyl seems important for the activity. The isopropenyl chain does not necessarily have to be opened: when attached to ring A as a bridge (HU-1009), the activity is even better.

We expected HU-1010, which is methylated on the quinone ring, to be more stable than HU-331, and as a result more active in bioassays. When dimers are formed from HU-331, its solution turns from yellow to violet (the process usually takes less than 12 h). HU-1010 indeed is much more stable than HU-331. While standing at room temperature for 48 h, the color is not changed, and the compound is still a monomer (by GC-MS, results not shown). To our surprise, however, the ability of this quinone to kill cancer cells is significantly much lower than that of HU-331. It is one of the least active compounds in our SAR. This presumably means that position C2′ of the quinone ring is needed not only to form the dimers, but also to bind to an assumed intracellular target. This assumption may also explain the lower activity of the DMH homolog of HU-331 (HU-333). This bulky side chain presumably hinders the C2 position, which is again presumably of importance for the molecule’s activity. However, the fact that this compound is able to kill cancer cells, though at much higher concentrations than HU-331, suggests that there is some additional mechanism of cancer cell death by cannabinoid quinones, not only topoisomerase II inhibition.

### 2.3. Antioxidant Evaluation

**Figure 3:**
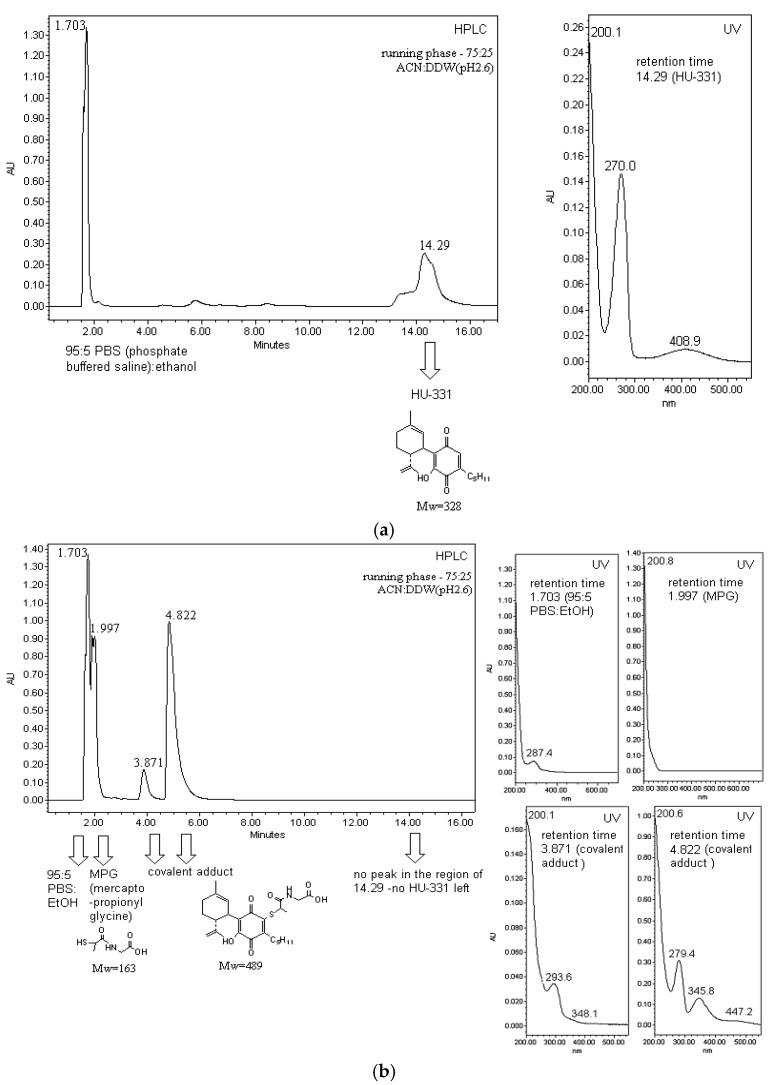
HPLC analysis of MPG binding to HU-331: (**a**) HPLC-UV of HU-331; (**b**) HPLC-UV of HU-331 after the addition of MPG.

In our previous work on the mechanism of anticancer activity of the anticancer cannabinoid quinone HU-331, we noted that two antioxidants NAC and MPG interfere with HU-331-mediated cancer cell death [19]. No other antioxidants or iron chelators were active. Furthermore, HU-331 was found to possess no pro-oxidant activity [19]; thus, it was clear that NAC and MPG interference with the effect of HU-331 was not due to their antioxidant activity. Our hypothesis was that, possessing a sulfhydryl group, both these compounds can bind directly to the quinone ring of HU-331. To prove this, HU-331, NAC, MPG and the mixtures of HU-331+NAC, HU-331+MPG were injected to an HPLC coupled to a UV detector. We assumed that if there is no covalent binding between HU-331 and NAC/MPG, in the mixture of HU-331+NAC/MPG, the peaks corresponding to the peaks of HU-331 and NAC/MPG will be seen at their original retention times, and with their original UV spectra. If there is a covalent binding, we will see some new peaks, and no peak of HU-331 (as an excess of NAC/MPG was added to the mixture). HU-331 is a very lipophilic molecule. Using C18 reversed phase HPLC column and a mobile phase of 75:25 acetonitrile:water (brought to pH2.6 with phosphoric acid), its peak appears at 14.29 min. Although it is quite problematic to dissolve large concentrations of HU-331 in PBS, it was used as a solvent, as it is used in the cell culture, where the anticancer effect of HU-331 can be seen. Being a quinone, HU-331 possesses a specific UV absorbance pattern, with peaks at 270 and 408.9 nm (Figure 3a).

MPG (mercaptopropionyl glycine), which possesses a strong antioxidant activity, is a very polar molecule. Using C18 reversed phase HPLC column and the mobile phase of 75:25 acetonitrile:water (pH2.6 with phosphoric acid), its peak appears at 1.997 min, immediately after the peak of PBS (phosphate buffered saline). PBS was used as a solvent, as it is used in the cell culture, where the effect of MPG can be seen (MPG interferes with the HU-331-mediated cancer cell death). MPG has no any UV absorbance. 

**Figure 4:**
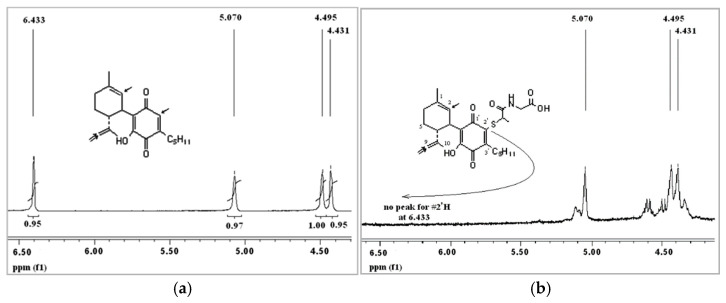
^1^H-NMR analysis of MPG binding to HU-331: (**a**) expansion of region 4–6.5 ppm of HU-331 H1-NMR; (**b**) expansion of region 4–6.5 ppm of HU-331 ^1^H-NMR after the addition of MPG.

When MPG and HU-331 are mixed together in the PBS and left for 15 min, there is no peak of HU-331 left in the chromatogram. Instead, two new peaks appear (at 3.871 and 4.822), which correspond to the covalent adducts between HU-331 and MPG (Figure 3b). The hypotheses that the covalent adducts are formed is supported by three observations: 1. the peak of HU-331 disappears; 2. the new peaks are much more polar (and thus appear much faster during the chromatogram run, which is logical as a very polar MPG is now covalently bound to the lipophilic HU-331, making the whole new molecule much more polar); and 3. the UV spectrum of these peaks is different from both HU-331 and MPG UV spectra. When peaks of the covalent adducts from HPLC are collected and injected into LCQ-MS, the molecular weight obtained is 488, which corresponds to 328 (HU-331) + 163 (MPG) −2 hydrogens at the place of attachment −1 for negative MS. All this supports the hypothesis that MPG interferes with HU-331-mediated cell death, not because it is an antioxidant, but because it binds covalently to HU-331, and probably blocks the position with which HU-331 would bind to its intracellular target (topoisomerase II). ^1^H-NMR of HU-331 further supports the covalent adduct theory: in the aromatic region, the peak corresponding to C2 hydrogen can be clearly seen for pure HU-331(6.433 ppm), and its integration is 1, exactly as the integration of the peak corresponding to C2 hydrogen on ring A (5.070 ppm) and each of the peaks of #C9 hydrogens (4.495 and 4.431 ppm) (Figure 4a). The NMR of covalent adducts MPG+HU-331, while still showing peaks at 5.070, 4.495 and 4.431 ppm (corresponding to hydrogens on C2 and C9 respectively), show the disappearance of the peak of the quinone hydrogen (peak at 6.433 ppm), indicating that the binding of MPG to HU-331 occurs at position C2 of the quinone ring (which in HU-331 is occupied by the quinone hydrogen) (Figure 4b). The assumption that the binding occurs at the quinone ring is supported also by the strong change in the UV absorbance from the original one of HU-331. The same results were obtained for NAC (not shown).

## 3. Discussion

The activity of cannabinoid quinones as anticancer compounds was as follows: 

In the Jurkat cell line—HU-395, HU-1007, HU-1009, HU-331, HU-1005, HU-396 and HU-1003 are more potent than HU1006, HU-1002, HU-1008, HU-1004, HU-1010, HU-333, HU1001.

In the Raji cell line—HU-1007, HU-1009, HU-395, HU-331, HU-396, HU-1005 and HU-1003 are more potent than HU-1002, HU-1006, HU-333, HU-1008, HU-1004, HU-1010, HU-1001.

**Figure 5:**
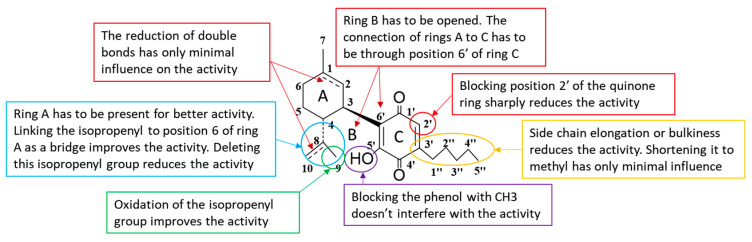
The summary of cannabinoid quinones structure-activity relationship (SAR).

From the structure-activity relationship in cannabinoid quinones series, the following understandings regarding the influence of different parts of the molecule on its anticancer activity can be drawn (also summarized in Figure 5):

- The double bonds on ring A have only a minor effect on the anticancer activity.
- The position at which rings A and C are attached one to another is of importance—ring C has to be attached through the “normal”, CBD-like position.
- The hydroxyl on ring C does not have to be free.
- Ring B has to be open.
- Ring A is of importance for the anticancer activity.
- The isopropenyl attached to position C4 of ring A is quite important for the anticancer activity; when absent the activity drops; the connection of isopropenyl to ring A as a bridge further improves the activity, as well as oxidation of the isopropenyl group attached to this ring.
- Position C2 of ring C is crucial for anticancer activity. If blocked or hindered, the activity sharply drops. This is probably a position through which a molecule binds to its intracellular target.

The importance of position C2’ of the quinone ring for the anticancer activity was first understood when a derivative methylated in this position, HU-1011, failed to kill cancer cells. The dimethylheptyl derivative, HU-333, is less active than HU-331. It is possible that the long DMH side chain sterically hinders position C2’, which is thus less available for reaction with intracellular targets. The antioxidants, MPG and NAC, which are able to inhibit the anticancer activity of HU-331, bind directly to the molecule in this position, as shown by HPLC coupled to UV and MS methods and NMR, further proving the importance of position C2’. 

This SAR study and results are based on antiproliferative activities on Raji and Jurkat cancer cell lines. This gives quite a clear view of the anticancer activity, though it has to be kept in mind that there is a possibility that the SAR might change when tested on different cancer cell lines, and of course this SAR is irrelevant for other activities (binding to CB1/CB2, PPARγ agonism or other possible targets). It would be very interesting and important to check the SAR on additional activities of these molecules in the next stage of the research. 

In summary, it seems that the topoisomerase-inhibiting activity (which needs the C2’ position to be free) and the PPARγ agonism (for which this position is not important and can be blocked) can be present in parallel in these molecules. Presumably, activity fine-tuning can be done by leaving this position open (thus shifting the activity towards topo II) or blocking it (thus shifting the activity more towards PPARγ).

The research on cannabinoid quinones is far from complete, and the possibility of targeting various diseases by acting on different mechanisms opens new horizons. 

## 4. Materials and Methods

### 4.1. NMR Spectroscopy

NMR data were collected on Varian Unity Inova 300 MHz spectrometer using the standard pulse sequences and processed with Agilent software.

### 4.2. Mass Spectrometry

The samples were analyzed by GC-MS in a Hewlett-Packard G1800 A GCD system with HP-5971 gas chromatograph with an electron ionization detector. Ultra-low-bleed 5%-phenyl capillary column (28 m × 0.25 mm (i.d.) × 0.25 μm film thickness) based on diphenyl methylsiloxane chemistry (HP-5MS; Agilent Technologies) was used.

### 4.3. Chemical Synthesis

All chemical reagents were purchased from Sigma-Aldrich (Rehovot, Israel). Organic solvents were purchased from Bio-Lab (Jerusalem, Israel)). CBD, cannabigerol (CBG) and cannabidiolic acid (CBD acid) were extracted from Lebanese-grown hashish. For column chromatography we used Sigma-Aldrich (Rehovot, Israel) silica gel, 60 A°. The syntheses were done as previously described for cannabinoid quinones [18] with slight modifications:

CBD (1.0 g, 3.18 mmole) was dissolved in 90 mL petroleum ether (40–60 °C bp) and 5% KOH_aq_ in ethanol (10 mL, 8.77 mmole), and a few drops of DMSO were added. The reaction mixture was stirred at 0 °C in O_2_ atmosphere for 3 h, after which 5% HCl (25 mL) was poured into it. The organic layer was washed with sodium bicarbonate and water and dried (MgSO_4_). Removal of the solvent under reduced pressure yielded a glassy oil (1.1 g). The brownish oil obtained was purified by column chromatography using a petroleum ether:diethyl ether (95:5) solution, and crystallized from pentane (50% yield).

HU-395: ^1^H-NMR (d6- DMSO): δ10.67 (s, 1H), 6.415 (t, 1H), 5.40 (s, 1H), 3.45 (m, 1H), 2.50 (t, 2H), 2.10, (m, 1H), 1.79 (s, 3H), 1.65 (m, 2H), 1.54 (m, 2H), 1.38 (m, 5H), 0.89 (m, 9H). *m*/*z* 330. 100% pure by GC-MS, brown crystals, m.p. 52–53 °C.

HU-396: ^1^H-NMR (d6- DMSO): δ 10.67 (s, 1H), 6.37 (t, 1H), 2.81 (m, 1H), 2.50 (t, 2H), 1.83 (m, 1H), 1.55 (s, 3H), 1.51 (m, 2H), 1.48 (m, 5H), 1.36 (m, 7H), 0.87 (m, 9H). m/z 332. 98.0% pure by GC-MS, brown oil.

HU-1001: ^1^H-NMR (d6- DMSO) δ 10.68 (s, 1H), 6.84 (s, 1H), 5.45 (s, 1H), 5.11 (s, 1H), 4.92 (s, 1H), 3.01 (m, 1H), 2.52 (m, 1H), 2.2 (m, 2H), 2.12 (m, 3H), 1.85 (m, 2H), 1.84 (s, 3H), 1.55 (s, 3H) 1.45–1.35 (m, 7H), 0.91 (t, 3H). m/z 328. 99.1% pure by GC-MS, brown oil.

HU-333: ^1^H-NMR (*d*_6_- DMSO): δ 10.67 (s, 1H) 6.70 (s, 1H), 5.238 (s, 1H), 4.92 (s, 1H), 4.45 (s, 1H), 2.87 (m, 1H), 2.17 (m, 1H), 1.98 (m, 1H), 1.85 (m, 10H), 1.22 (m, 10H), 1.05 (m, 2H), 0.83 (t,3H); *m*/*z* 384. 98.7% pure by GC-MS, reddish-brown oil.

HU-1002: 1H NMR (d_6_- DMSO): δ 10.68 (s, 1H), 6.72 (s, 1H), 5.13 (s, 1H), 4.56 (m, 2H), 3.72 (d, 1H), 2.76 (m, 1H), 2.20 (m, 1H), 2.00 (m, 1H), 1.72 (m, 1H), 1.67 (s, 3H), 1.58 (s, 3H), 1.28 (s, 9H). *m*/*z* 314. 99.2% pure by GC-MS, yellowish-brown oil.

HU-1003: ^1^H-NMR (d_6_- DMSO): δ 10.69 (s, 1H), 6.71 (s, 1H), 5.36 (s, 1H), 4.47 (s, 1H), 4.40 (s, 1H), 3.57 (s, 1H), 2.52 (s, 3H), 2.26 (m, 1H), 2.03 (m, 2H), 2.01 (m, 1H), 1.76 (m, 1H), 1.72 (t, 1H), 1.68 (s, 3H); *m*/*z* 272. 98.8% pure by GC-MS, dark yellow oil.

HU-1004: ^1^H-NMR (d6- DMSO): δ 10.68 (s, 1H), 6.71 (s, 1H), 5.38 (s, 1H), 5.01 (s, 1H), 4.92 (s, 1H), 3.21 (m, 1H), 2.42 (s, 3H), 2.06 (m, 1H), 2.01 (m, 2H), 1.87 (m, 1H), 1.75 (s, 3H), 1.63 (s, 3H), 1.55(m, 1H); *m*/*z* 272. 99.0% pure by GC-MS, dark yellow oil. 

HU-1005: ^1^H-NMR (d_6_- DMSO): δ 10.68 (s, 1H), 6.41 (s, 1H), 5.16 (s, 1H), 4.47 (s, 1H), 4.40 (s, 1H), 3.8 (s, 1H), 3.72 (s, 3H), 2.96 (td, 1H), 2.52 (m, 2H), 2.17 (m, 1H), 2.13 (m, 3H), 2.01 (m, 1H), 1.76 (m, 2H), 1.68 (s, 3H), 1.54 (m, 6H), 1.36 (m, 4H), 0.89 (t, 3H) *m*/*z* 342. 98.5% pure by GC-MS, brown oil.

HU-1006: ^1^H-NMR (d6- DMSO): δ 10.68 (s, 1H), 6.25 (s, 1H), 5.25 (m, 1H), 5.06 (m, 1H), 4.84 (s, 1H), 2.68 (d, 2H), 2.50 (t, 2H), 2.1 (m, 1H), 1.85 (s, 3H), 1.69 (s, 3H), 1.62 (s, 3H), 1.54 (m, 2H), 1.34 (m, 4H), 0.91 (t, 3H). *m*/*z* 330. 99.7% pure by GC-MS, light brown crystal, m.p. 44–45 °C.

HU-1007: 1H NMR (d_6_- DMSO): δ 10.68 (s, 1H), 9.29 (s, 1H) 6.73 (s, 1H), 6.05 (s, 1H), 5.76 (s, 1H), 5.14 (s, 1H), 3.77 (m, 1H), 2.99 (m, 1H), 2.54 (m, 2H), 2.13 (m, 1H), 1.950 (m, 2H), 1.770 (m, 1H), 1.62 (s, 3H), 1.41 (m, 2H), 1.29 (m, 4H), 0.87 (t, 3H). 98.8% pure by GC-MS, brown oil.

HU-1008: ^1^H-NMR (d6- DMSO): δ 10.68 (s, 1H), 6.415 (t, 1H), 5.40 (s, 1H), 3.01 (m, 1H), 2.2 (t, 2H), 2.12 (m, 3H), 2.02 (m, 1H), 1.85 (m, 2H), 1.65 (s, 3H) 1.40 (m, 4H), 0.91 (t, 3H); *m*/*z* 288. 99.2% pure by GC-MS, brown oil.

HU-1009: ^1^H-NMR (d6- DMSO): δ 10.68 (s, 1H), 6.70 (s, 1H), 5.69 (s, 1H), 3.94 (m, 1H), 2.75 (m, 1H), 2.31 (t, 2H), 2.19(m, 1H), 1.86 (m, 1H), 1.69(m, 1H), 1.62 (s, 3H), 1.55 (m, 2H), 1.34 (m, 4H), 1.15 (s, 6H), 0.89 (t, 3H). *m*/*z* 328. 98.5% pure by GC-MS, reddish-brown oil. 

HU-1010: ^1^H-NMR (d6- DMSO): δ 10.68 (s, 1H), 7.05 (s, 1H), 5.15 (s, 1H), 4.54 (m, 2H), 3.74 (s, 1H), 2.75 (m, 1H), 2.46 (t, 2H), 2.11 (s, 3H), 2.02 (m, 1H), 1.78 (s, 3H), 1.76 (m, 2H), 1.68 (m, 1H), 1.63 (s, 3H), 1.55 (m, 2H), 1.3 (m, 4H), 0.89 (t, 3H); *m*/*z* 342. 98.4% pure by GC-MS, brown oil.

### 4.4. Cell Culture

Raji and Jurkat cells were suspended in RPMI 1640 medium, supplemented with 20% heat-inactivated fetal calf serum, 2 mM l-glutamine, 100 U/mL penicillin and 0.01 mg/mL streptomycin at 37 °C in a 5% CO2 humidified atmosphere. Other cell lines were suspended in RPMI 1640 medium, supplemented with 10% heat-inactivated fetal calf serum, 2 mM l-glutamine, 100 U/mL penicillin and 0.01 mg/mL streptomycin at 37 °C in a 5% CO2-humidified atmosphere.

### 4.5. Cell Proliferation Test

Aliquots of suspensions of cancer cells were dispensed at 200 μL volumes into wells of 96-well tissue culture plates at densities of 0.02 × 10^6^ cells/well. Various concentrations of cannabinoid quinones were introduced into the wells, and their efficacy was tested three days after initiation of the cultures, using the 3-(4,5-dimethylthiazol-2-yl)-2,5-diphenyltetrazolium bromide (MTT) assay [18]. The principle of this assay is that cells which survive following exposure to various compounds can reduce MTT to a dark-colored formazan, while dead cells are incapable of doing so. The assay was performed as described previously. In each MTT assay, every concentration of the cytotoxic substance was tested in five replicates in microplate wells. Assays with every cell line were carried out in two-to-three repeated experiments. The inhibitory effect of various compounds was calculated as percentage inhibition in comparison with the values obtained in untreated wells to which the vehicle (ethanol 0.5%) was added.

### 4.6. Statistical Analyses

Statistical analysis was performed with GraphPad Prism software. The IC_50_ values were calculated by the nonlinear regression a four-parameter dose-response curve equation: log(inhibitor) vs. response with variable slope. This model does not assume a standard slope, but rather fits the Hill Slope from the data. The IC50 values of different groups were compared by 1-way ANOVA and post hoc analysis by Dunnett’s test. * *p*-value < 0.05, ** *p*-value < 0.01 and *** *p*-value < 0.001 when comparing to HU-331 were used as a control group.

## Figures and Tables

**Table 1:** IC_50_ values of cannabinoid quinones on the Raji and Jurkat cell lines.

Comp. Name	Raji (IC_50_, µM)	Jurkat (IC_50_, µM)
HU-331	0.603 ± 0.047	0.470 ± 0.057
HU-395	0.227 ± 0.037	0.154 ± 0.036
HU-396	0.617 ± 0.035	0.549 ± 0.066
HU-1001	25.14 ± 0.962 ***	39.07 ± 3.102 ***
HU-333	5.859 ± 0.794 **	17.54 ± 3.025 ***
HU-1002	2.044 ± 0.125 *	6.373 ± 0.386 **
HU-1003	0.820 ± 0.022	0.772 ± 0.075
HU-1004	11.01 ± 1.182 ***	9.984 ± 1.016 ***
HU-1005	0.634 ± 0.073	0.499 ± 0.045
HU-1006	4.270 ± 0.149 *	4.301 ± 0.168 *
HU-1007	0.179 ± 0.01	0.203 ± 0.041
HU-1008	8.474 ± 0.982 ***	9.229 ± 1.03 ***
HU-1009	0.220 ± 0.038	0.245 ± 0.076
HU-1010	34.01 ± 2.665 ***	17.31 ± 0.506 ***

Data presented as mean ± SEM. * *p*-value < 0.05, ** *p*-value < 0.01 and *** *p*-value < 0.001 when comparing to HU-331.

## Data Availability

Data is contained within the article. The data presented in this study are available in this article, and can be sent in more detail on request from the corresponding author.

## Sample Availability

Samples of the compounds are unavailable from the authors.
